# Altered autonomic control of heart rate variability in the chronically hypoxic fetus

**DOI:** 10.1113/JP275659

**Published:** 2018-04-29

**Authors:** C. J. Shaw, B. J. Allison, N. Itani, K. J. Botting, Y. Niu, C. C. Lees, D. A. Giussani

**Affiliations:** ^1^ Department of Physiology, Development and Neuroscience University of Cambridge Cambridge UK; ^2^ Cambridge Cardiovascular Research Initiative Addenbrooke's Hospital Cambridge UK; ^3^ Institute of Reproductive and Developmental Biology Imperial College London London UK; ^4^ Department of Obstetrics and Gynaecology University Hospitals Leuven Leuven Belgium

**Keywords:** Fetal heart rate, Fetal heart rate variability, Sympathetic nervous system, Fetal Growth Restriction, Intrauterine hypoxia

## Abstract

**Key points:**

- Fetal heart rate variability (FHRV) has long been recognised as a powerful predictor of fetal wellbeing, and a decrease in FHRV is associated with fetal compromise. However, the mechanisms by which FHRV is reduced in the chronically hypoxic fetus have yet to be established.
- The sympathetic and parasympathetic influences on heart rate mature at different rates throughout fetal life, and can be assessed by time domain and power spectral analysis of FHRV.
- In this study of chronically instrumented fetal sheep in late gestation, we analysed FHRV daily over a 16 day period towards term, and compared changes between fetuses of control and chronically hypoxic pregnancy.
- We show that FHRV in sheep is reduced by chronic hypoxia, predominantly due to dysregulation of the sympathetic control of the fetal heart rate. This presents a potential mechanism by which a reduction in indices of FHRV predicts fetuses at increased risk of neonatal morbidity and mortality in humans.
- Reduction in overall FHRV may therefore provide a biomarker that autonomic dysregulation of fetal heart rate control has taken place in a fetus where uteroplacental dysfunction is suspected.

**Abstract:**

Although fetal heart rate variability (FHRV) has long been recognised as a powerful predictor of fetal wellbeing, the mechanisms by which it is reduced in the chronically hypoxic fetus have yet to be established. In particular, the physiological mechanism underlying the reduction of short term variation (STV) in fetal compromise remains unclear. In this study, we present a longitudinal study of the development of autonomic control of FHRV, assessed by indirect indices, time domain and power spectral analysis, in normoxic and chronically hypoxic, chronically catheterised, singleton fetal sheep over the last third of gestation. We used isobaric chambers able to maintain pregnant sheep for prolonged periods in hypoxic conditions (stable fetal femoral arterial $P_{O_{2}}$ 10–12 mmHg), and a customised wireless data acquisition system to record beat‐to‐beat variation in the fetal heart rate. We determined *in vivo* longitudinal changes in overall FHRV and the sympathetic and parasympathetic contribution to FHRV in hypoxic (*n* = 6) and normoxic (*n* = 6) ovine fetuses with advancing gestational age. Normoxic fetuses show gestational age‐related increases in overall indices of FHRV, and in the sympathetic nervous system contribution to FHRV (*P* < 0.001). Conversely, gestational age‐related increases in overall FHRV were impaired by exposure to chronic hypoxia, and there was evidence of suppression of the sympathetic nervous system control of FHRV after 72 h of exposure to hypoxia (*P* < 0.001). This demonstrates that exposure to late gestation isolated chronic fetal hypoxia has the potential to alter the development of the autonomic nervous system control of FHRV in sheep. This presents a potential mechanism by which a reduction in indices of FHRV in human fetuses affected by uteroplacental dysfunction can predict fetuses at increased risk.

## Key points

- Fetal heart rate variability (FHRV) has long been recognised as a powerful predictor of fetal wellbeing, and a decrease in FHRV is associated with fetal compromise. However, the mechanisms by which FHRV is reduced in the chronically hypoxic fetus have yet to be established.
- The sympathetic and parasympathetic influences on heart rate mature at different rates throughout fetal life, and can be assessed by time domain and power spectral analysis of FHRV.
- In this study of chronically instrumented fetal sheep in late gestation, we analysed FHRV daily over a 16 day period towards term, and compared changes between fetuses of control and chronically hypoxic pregnancy.
- We show that FHRV in sheep is reduced by chronic hypoxia, predominantly due to dysregulation of the sympathetic control of the fetal heart rate. This presents a potential mechanism by which a reduction in indices of FHRV predicts fetuses at increased risk of neonatal morbidity and mortality in humans.
- Reduction in overall FHRV may therefore provide a biomarker that autonomic dysregulation of fetal heart rate control has taken place in a fetus where uteroplacental dysfunction is suspected.

## Introduction

Antenatal electronic monitoring of fetal heart rate variability (FHRV) is an important clinical tool to assess the fetal condition, as authoritatively described over many years by Parer (Fox *et al*. 2000). It is routinely used to assess fetal wellbeing in pregnancies affected by uteroplacental dysfunction and profound, prolonged reductions in FHRV are thought to represent acute fetal compromise (Turan *et al*. 2007; Serra *et al*. 2008). A persistent reduction in short term variation (STV) below 3 ms in the antenatal period, within 24 h of delivery, is predictive of an increased risk of metabolic acidosis in the neonate at birth, and early neonatal death (Serra *et al*. 2008). As such, STV, as part of computerised cardiotocography (CTG) examination, is recommended for antenatal surveillance of human fetuses with suspected uteroplacental dysfunction and chronic fetal hypoxia to detect acute fetal distress (Royal College of Obstetricians and Gynaecologists, 2013). However, the physiological basis underlying this reduction in FHRV remains unclear.

The fetal heart rate (FHR) fluctuates under the influence of basal sympathetic and parasympathetic tone, the central nervous system, chronotropic hormones and different fetal sleep states (Nijhuis *et al*. 1982), and it also has some intrinsic variability (Kimura *et al*. 1996). The sympathetic and parasympathetic influences on FHRV mature at different rates throughout gestation (Walker *et al*. 1979), and their relative contributions can be assessed by indirect indices, time domain and power spectral analysis. Assessment of these indices mandates the insertion of a fetal electrode, arterial catheter or flow probe, which has significantly limited human studies in this field and thereby clinical application (Peters *et al*. 2004). Recently, fetal magnetic resonance imaging (MRI) has been used to acquire FHR on a sufficiently accurate beat‐to‐beat basis to allow such analysis, termed a fetal magnetocardiogram (fMCG) (Ferrario *et al*. 2009; Sriram *et al*. 2013).

Time domain and power spectral indices of FHRV need to be separated by fetal behavioural state. Fetal behavioural states (1F–4F) relate to quiet and active sleep (1F = quiet sleep, 2F = active sleep, 3F = fetal breathing or thumb sucking, 4F = gross fetal movements). The correlation between oscillation bandwidth of heart rate variability and frequency of accelerations of fetal heart rate is sufficiently strong that different states can be determined by visual identification of heart rate patterns alone (Schaffer *et al*. 2009), an established method previously used in this field (van Laar *et al*. 2009). Ultrasound‐CTG studies (Henson *et al*. 1984), fMCG studies (Sriram *et al*. 2013), as well a fetal electrocortical studies in chronically instrumented sheep (Keen *et al*. 2011) demonstrate that the relative time spent in each behavioural state is unchanged between healthy and chronically hypoxic fetuses.

A recent elegant study investigated the normal evolution of frequency power spectra over the last third of gestation in chronically instrumented, healthy singleton fetal sheep at 0.6, 0.7 and 0.8 of gestation. The study reported that total spectral power increased with advancing gestational age, and that sympathetic dominance of FHRV was more evident in active than quiet sleep (Koome *et al*. 2014a). Similar studies in chronically hypoxic sheep fetuses have not been reported to date. Progress in this field has been hampered in part by the inability to record continuous cardiovascular function in the fetus during the period of chronic fetal hypoxia. We have now designed and created isobaric hypoxic chambers able to maintain pregnant sheep for prolonged periods of gestation under controlled, long‐term hypoxia (Brain *et al*. 2015). We have also established a wireless data acquisition system, able to record fetal cardiovascular signals from free‐moving ewes as the hypoxic pregnancy is developing (Brain *et al*. 2015; Allison *et al*. 2016). In this study, we have used these novel technologies to determine a longitudinal study of the development of FHRV, assessed by time domain and power spectral analysis, in normoxic and chronically hypoxic, chronically instrumented, singleton fetal sheep over the last third of gestation. The last third of gestation was chosen as the period of chronic hypoxia as this is the time of maximal fetal growth, a period known to be affected by chronic fetal hypoxia.

The study tested the hypothesis that hypoxia induced in the last third of gestation in sheep affects the autonomic regulation of the ontogeny of the fetal heart rate power spectrum, thereby contributing to a reduction in FHRV.

## Methods

### Ethical approval

All procedures were performed in accordance with the UK Animals (Scientific Procedures) Act 1986 and were approved by the Ethical Review Board of the University of Cambridge.

### Surgical preparation and post‐operative care

**Figure 1:**
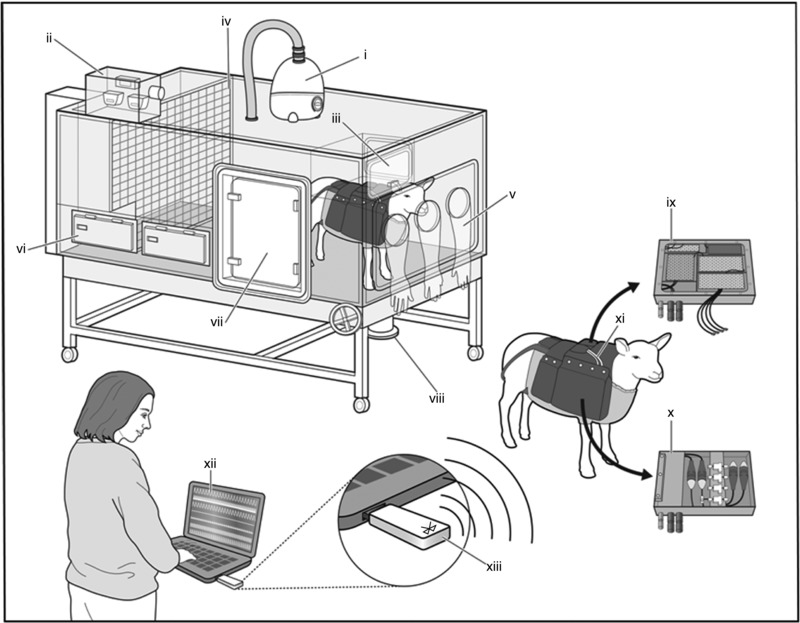
Isobaric hypoxic chambers and the CamDAS system Each chamber was equipped with an electronic servo‐controlled humidity cool steam injection system to return the appropriate humidity to the inspirate (i). Ambient $P_{O_{2}}$, $P_{CO_{2}}$, humidity and temperature within each chamber were monitored via sensors (ii). For experimental procedures, each chamber had a double transfer port (iii) to internalise material and a manually operated sliding panel (iv) to bring the ewe into a position where daily sampling of blood could be achieved through glove compartments (v). Each chamber incorporated a drinking bowl on continuous water supply and a rotating food compartment (vi) for determining food intake. A sealed transfer isolation cart could be attached to a side exit (vii) to couple chambers together for cleaning. The CamDAS system was contained in a custom‐made sheep jacket able to hold the data acquisition system box (ix) in one side pouch and a box containing the pressure connectors (x) in the other. Cables (xi) connected the two boxes together and also to two battery packs able to power the system for 24 h. Measurements made using the data acquisition were transmitted wirelessly via Bluetooth (xiii) to a laptop kept outside the chamber room (xii) on which it was possible to view continuous recordings of the maternal and fetal cardiovascular data (reproduced with permission, Allison *et al*. 2016).

Twelve pregnant Welsh mountain sheep carrying singleton fetuses were used in the study. In brief, using the protocol described in Allison *et al*. (2016), at 117 ± 1 days of gestation (0.8 of gestation; term ∼150 days), general anaesthesia was induced using 1.5–2.5 mg kg^−1^ i.v. alfaxalone (Jurox Ltd, Malvern, UK). The ewe was intubated with a cuffed endotracheal tube and anaesthesia was maintained by inhalation of 1.5% isoflurane in oxygen. Under aseptic surgical conditions, an arterial catheter was inserted into the fetal femoral artery and advanced into the descending aorta. Another catheter was placed in the amniotic cavity. Following surgery, ewes were housed in individual floor pens with a 12 h light–dark cycle with *ad libitum* access to hay, nuts and water. After 5 days post‐operative recovery, ewes and fetuses were randomly allocated to chronic normoxia (*n* = 6) or chronic hypoxia (*n* = 6). Chronically hypoxic animals were housed in bespoke isobaric hypoxic chambers (Fig. 1) for 2 days prior to the initiation of hypoxia, and remained in these chambers for a further 10 days under hypoxic conditions, before being returned to the individual floor pens and normoxic conditions. Hypoxia was induced incrementally over the first 24 h, then maintained at 10% inspired oxygen for the remainder of the experimental protocol, the full details of which have been previously described (Brain *et al*. 2015; Allison *et al*. 2016). Pregnancies allocated to the chronic normoxia group were housed in a barn in floor pens with the same floor area as that of the hypoxic chambers. Both the chronic normoxia and hypoxia groups of ewes were fed daily the same bespoke maintenance diet made up of concentrate pellets and hay (40 g nuts kg^–1^ and 3 g hay kg^–1^; Manor Farm Feeds Ltd, Oakham, UK) to facilitate the monitoring of food intake. Ambient room temperature was maintained between 20 and 24°C in both the hypoxic chambers and the normoxic floor pens. At the end of the experimental protocol the ewe and fetus were killed under Schedule 1 of the UK Animals (Scientific Procedures) Act 1986 using a slow i.v. injection into the maternal jugular vein of 120 mg kg^−1^ pentobarbitone sodium (Pentoject; Animalcare Ltd, York, UK).

### Fetal heart rate recording and identification of sleep state

The fetal arterial and amniotic catheters were connected to pressure transducers (ArgoTrans; Argon Medical Devices Inc., Frisco, TX, USA). This allowed measurement of continuous changes in fetal pulsatile arterial blood pressure. From this, the inter‐beat interval (equal to the time between systolic peaks) could be assessed and time domain and power spectral analyses calculated.

**Figure 2:**
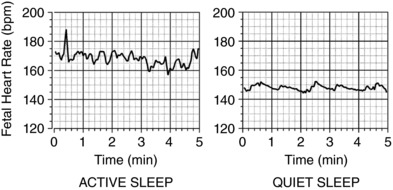
Fetal heart rate variability patterns A representative example of active sleep (left) and quiet sleep (right) categorised by visual identification.

Continuous physiological values of fetal heart rate were recorded using a customised data acquisition system, CamDAS (Fig. 1), from the immediately post‐operative period to the end of the experiment (Allison *et al*. 2016). These data were converted into absolute physiological values by IDEEQ data recording software at a sampling rate of 500 kHz (IDEEQ, Maastricht Instruments, Maastricht, The Netherlands), and was available thereafter for offline data analysis. Fetal heart rate was sampled daily in 5 min blocks spaced every 30 min between midnight and 06.00 h. This time frame was selected as the period when ewes were least likely to be disturbed by human activity in the research facility. Therefore, a total of 30 min of FHR per 24 h was available for further analysis. Five‐minute epochs were chosen for analysis as this is the established recommended length of time over which standard deviation of normal to normal R‐R intervals (SDNN) should be calculated, and does not affect the calculation of low frequency (LF), high frequency (HF), total power or STV (Malik *et al*. 1996). Mean values for sequential 2.5 s epochs throughout the recording period were generated for cardiovascular data using Labchart 7 Pro (ADInstruments Pty Ltd, Bella Vista, NSW, Australia) and used to create a CTG of fetal heart rate (Fig. 2). This allowed visual interpretation of the fetal heart rate pattern. Segments were then visually identified as FHR patterns A–D (correlating to fetal behaviour states 1F–4F); quiet sleep was defined as FHR pattern A and active sleep as FHR pattern B. Records classified as FHR pattern A or B were included; FHR patterns C and D were excluded. If no suitable FHR pattern was acquired, re‐sampling was performed within the same time period for that day.

### Fetal heart rate variability analysis

Time domain and power spectral analysis was performed in Labchart 7 Pro (ADInstruments). The time between successive systolic peaks in arterial blood pressure were used to define the R–R inter‐beat interval. Based on previously published literature the frequency boundaries used were: very low frequency (VLF) 0–0.04 Hz, LF 0.04–0.15 Hz and HF 0.15–0.4 Hz (Min *et al*. 2002; van Laar *et al*. 2009; Koome *et al*. 2014b). Mean daily values for average heart rate, SDNN, root mean square of the successive differences (RMSSD), absolute and normalised LF and HF, LF/HF ratio, absolute VLF and total power were calculated daily from the categorised 5 min samples. The baseline value quoted represents an average of 72 h (days 3–5 post‐surgery). STV was calculated using R–R intervals: first, average heart rate was used to calculate the baseline R–R value; second, the baseline R–R value was used to exclude values representing accelerations (≥10 beats min^−1^ above baseline for ≥15 s) or decelerations (≤20 beats min^−1^ below baseline for ≥30 s) as these are excluded by STV calculation software (Pardey *et al*. 2002). The remaining values were averaged in 3.75 s (1/16th min) epochs and the mean difference between 16 sequential periods (1 min mean difference) was calculated as per published STV calculation algorithms (Street *et al*. 1991). Final STV values are the mean of the 5 min mean difference values. All these values were compared between normoxic and hypoxic pregnancies in quiet and active sleep states.

### Blood sampling regime and analysis

Samples of descending aortic fetal (0.3 ml) and maternal (1 ml) blood were taken daily. These were used to determine acid–base status, and partial pressures of oxygen and carbon dioxide (ABL5 Blood Gas Analyser, Radiometer, Copenhagen, Denmark), and haemoglobin and oxygen saturation of the blood (OSM3, Radiometer).

### Determination of hormone concentrations

An additional 1 ml of fetal arterial blood was taken after 5 days post‐operative recovery (baseline) and 2 days after removal from chronic hypoxia, or equivalent time in normoxic controls (day 13) for determination of plasma cortisol and catecholamine concentrations. Blood was collected into EDTA, centrifuged for plasma extraction and frozen at −80°C. The concentration of fetal cortisol in plasma was quantified using a commercially available cortisol indirect enzyme‐linked immunosorbent assay (ELISA) kit according to the product instructions (RE52061, IBL International, Hamburg, Germany). This assay has previously been validated for use in fetal ovine plasma (Kabaroff *et al*. 2006). Duplicate 20 μl plasma aliquots (undiluted) were taken from previously unthawed samples. The inter‐assay and intra‐assay coefficients of variation were 5.2% and 5.0%, respectively. The lower limit of detection was 3.2 ng ml^−1^. The concentrations of fetal noradrenaline and adrenaline in plasma were quantified using a commercially available ELISA kit according to product instructions (KA1877, Abnova, Taipei, Taiwan) which has been previously optimised and validated for ovine plasma in our laboratory (Brain *et al*. 2015). Duplicate 300 μl volumes of previously unthawed plasma were used to extract noradrenaline and adrenaline. For noradrenaline, the inter‐assay and intra‐assay coefficients of variation were 12.8% and 14.6%, respectively, and the lower limit of detection was 0.05 ng ml^−1^. For adrenaline, the inter‐assay and intra‐assay coefficients of variation were 9.7% and 15.7%, respectively, and the lower limit of detection was 0.01 ng ml^−1^.

### Statistical analyses

Values are expressed as mean ± SEM. Statistical analysis was performed in SPSS 22.0 (SPSS Inc., Chicago, IL, USA). Repeated measures (RM) two‐way ANOVAs were performed: if a significant interaction between gestational age and oxygenation was identified, *post hoc* Holm–Sidak tests were performed to assess the effect of oxygenation and *post hoc* Tukey's tests were performed to assess the change from baseline with advancing gestational age. Normality was assessed with the Shapiro–Wilk test and for normally distributed data means were compared with Student's *t* test. Statistical significance was accepted when *P *< 0.05.

## Results

### Maternal and fetal oxygenation, acid–base and endocrine status

**Figure 3:**
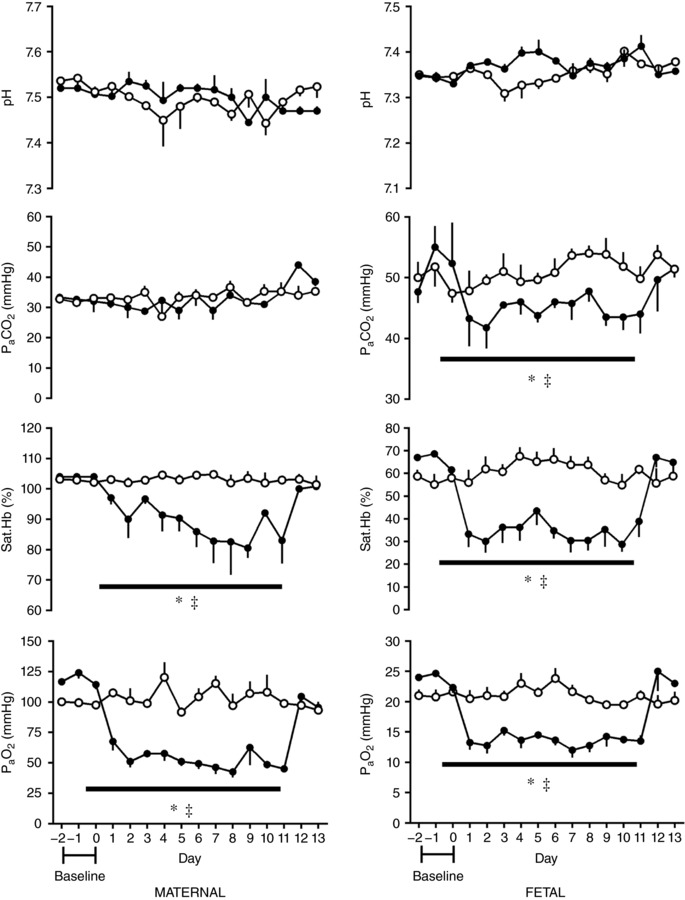
Maternal and fetal blood gas, acid–base status during chronic normoxia and hypoxia Values represent the mean ± SEM (*n* = 12) for arterial pH, partial pressure of arterial carbon dioxide (P aC O2) and oxygen ($P_{aO_{2}}$) and percentage oxygen saturation of haemoglobin (Sat.Hb). Chronic hypoxia, filled circles; chronic normoxia, open circles. Significant differences (*P* < 0.05): ^*^significant main effect of hypoxia compared with normoxia; ^‡^significant main effect of time in hypoxic pregnancy on each individual day 1–13 when compared to the baseline period (RM two‐way ANOVA with *post hoc* Holm–Sidak and Tukey's tests).

Baseline values for maternal and fetal pH, partial pressure of arterial oxygen ($P_{aO_{2}}$) and carbon dioxide (P aC O2) and the saturation of oxyhaemoglobin (Sat.Hb) were not different between groups prior to randomisation to chronic hypoxia or normoxia (Fig. 3), and were within normal ranges for Welsh mountain ewes at this gestational age (Fletcher *et al*. 2003, 2006). Daily maternal food intake at baseline was not different between groups: 1.3 ± 0.9 kg day^−1^ (to be assigned normoxia) and 1.1 ± 0.4 kg day^−1^ (to be assigned hypoxia) and maternal food intake was not affected by exposure to chronic hypoxia (Brain *et al*. 2015).

There was a significant fall in maternal and fetal $P_{aO_{2}}$ and Sat.Hb during the period in which the fraction of maternal inspired oxygen was reduced to 10% in the chronic hypoxia group; maternal and fetal oxygenation levels remained stable in the normoxic control group. There was no effect of time or oxygenation on maternal or fetal pH, and maternal P aC O2 was unaffected by time or treatment. However, fetal P aC O2 was reduced by the effect of chronic hypoxia (Fig. 3).

Baseline (122 ± 1 days gestational age (GA)) fetal cortisol, noradrenaline and adrenaline concentrations in plasma were not different between animals to be assigned to either chronic normoxic or chronic hypoxia treatment groups. There was a gestational age‐dependent rise in fetal plasma cortisol concentration by the time of the second sample (136 ± 1 days GA). However, the magnitude of the ontogenic increase in fetal plasma cortisol was not affected by whether the fetus had previously been exposed to chronic hypoxia or normoxia. There was no change in concentration of plasma catecholamines related to either gestational age or treatment group (Table 1).

**Table 1:** Ontogenic changes in fetal cortisol and catecholamines during chronic hypoxia and normoxia

	Normoxia		Hypoxia	
	Baseline	Day 13	Baseline	Day 13
Cortisol (ng ml^−1^)	11.9 ± 1.4	24.1 ± 1.8*	13.8 ± 1.8	32.2 ± 5.2*
Noradrenaline (ng ml^−1^)	1.1 ± 0.3	0.8 ± 0.1	0.7 ± 0.1	0.6 ± 0.1
Adrenaline (ng ml^−1^)	0.09 ± 0.02	0.07 ± 0.02	0.06 ± 0.01	0.07 ± 0.01

Values represent the mean ± SEM of plasma concentrations of fetal cortisol, noradrenaline and adrenaline at baseline (122 ± 1 days GA) and 2 days after the end of chronic hypoxia/normoxia (day 13, 136 ± 1 days GA). Significant differences (*P* < 0.05): ^*^significant effect of gestational age (RM two‐way ANOVA with *post hoc* Tukey's test).

### Effects on FHRV of gestational age and of chronic hypoxia

A total of 882 FHR records were analysed in this study, 107 (12%) during quiet sleep (FHR pattern A) and 477 (54%) during active sleep (FHR pattern B). The remainder of the FHR patterns (34%) were either C or D. This distribution of fetal sleep states is expected from the observed distribution of fetal behavioural states at these gestational ages (van Woerden & van Geijn, 1992). In the normoxic group, 381 of 505 FHR records were in either pattern A or B (75%); in the hypoxic group 501 of 702 (71%) were in either pattern A or B (χ^2^ test, *P* = 0.11). In the baseline state, prior to randomisation to chronic normoxia or hypoxia, there were no differences between the fetal absolute heart rate or any indices of fetal heart rate variability based on their future treatment group. There was no effect of sleep state on fetal heart rate between quiet and active sleep. However, there was an effect of sleep state at 0.8 of gestation observed in SDNN, STV, total power, LF, HF, VLF, LF/HF ratio, but not RMSSD (Table 2).

**Table 2:** Effect of sleep state on FHRV indices before onset of chronic normoxia or hypoxia

	Active sleep 0.8 gestational age	Quiet sleep 0.8 gestational age	*P* value
Fetal heart rate (beats min^−1^)	173 ± 1.5	175.6 ± 1.6	0.2625
Standard deviation of normal to normal R‐R intervals (SDNN) (ms)	12.5 ± 0.4	6.7 ± 0.3*	<0.001
Short term variation (STV) (ms)	4.7 ± 0.2	2.6 ± 0.1*	<0.001
Total power (ms^2^)	166 ± 11.1	6.2 ± 0.7*	<0.001
Low frequence (LF) power (ms^2^)	82.6 ± 7.4	8.2 ± 1.4*	<0.001
High frequency (HF) power (ms^2^)	19 ± 1.7	5.9 ± 0.6*	<0.001
LF/HF ratio	4.7 ± 0.5	1.5 ± 0.2*	<0.001
Root mean square of the successive differences (RMSSD) (ms)	8.3 ± 0.6	7.6 ± 0.8	0.5148

Values represent the mean ± standard error of the mean (*n* = 12) for fetal heart rate and indices of fetal heart rate variability in quiet and active sleep. Significant differences (*P* < 0.05): ^*^significant effect of sleep state (Student's *t* test).

**Figure 4:**
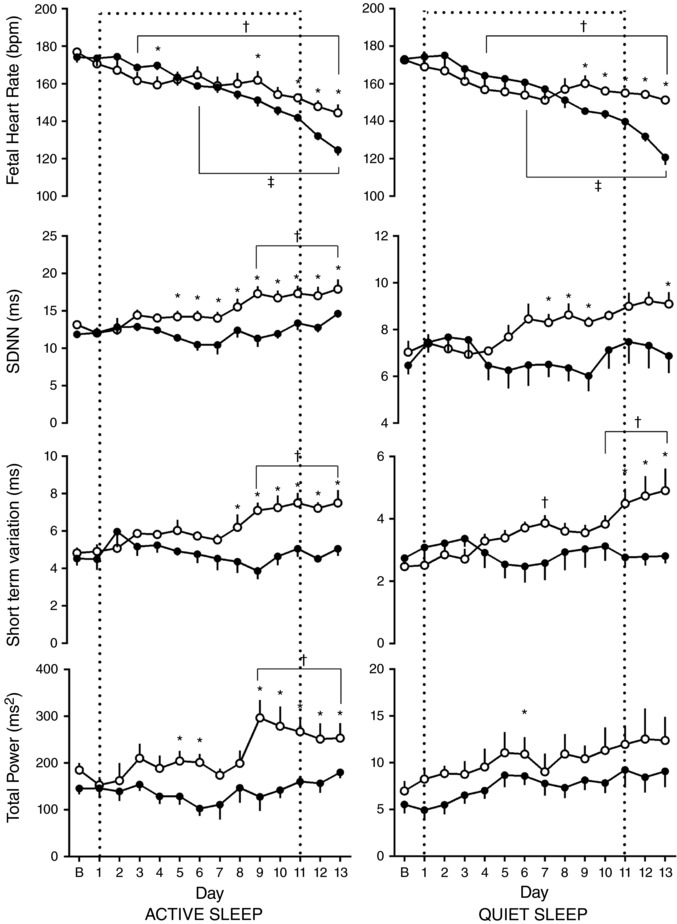
Ontogenic changes in fetal heart rate and variability during chronic normoxia and hypoxia Values represent the mean ± SEM (*n* = 12) for absolute fetal heart rate, SDNN, short term variation (STV) and total power in quiet and active sleep. Period of chronic hypoxia (filled circles) or normoxia (open circles) indicated by dashed box. ‘B’ represents an average of values taken in the 72 h period −2 to 0. Significant differences (*P* < 0.05): ^*^significant effect of oxygenation between treatment groups; ^†^significant effect of gestational age in normoxic pregnancy on each individual day 1–13 when compared to the baseline ‘B’ period; ^‡^significant effect of gestational age in hypoxic pregnancy on each individual day 1–13 when compared to the baseline ‘B’ period (RM two‐way ANOVA with *post hoc* Tukey's and Holm–Sidak tests).

In both quiet and active sleep states, there was a decrease in average fetal heart rate with increasing gestational age. However, this fall was greater in the chronically hypoxic group than in normoxic pregnancies (Fig. 4). There was a corresponding gestational age‐dependent increase in FHRV in the normoxic group. Measured by SDNN and STV, this reached significance 9 days after baseline in active sleep. In normoxic pregnancy in quiet sleep, STV first showed a significant rise 7 days after baseline, and was consistently elevated 10 days after baseline. However, the trend to increase observed in SDNN did not reach significance over the period assessed. This increase in overall FHRV was not observed in the chronic hypoxia group, in either quiet or active sleep, measured by SDNN or STV. An oxygenation‐dependent difference between groups in STV and SDNN was observed, although this occurred earlier in active sleep than quiet sleep (Fig. 4). In normoxic pregnancies in active sleep there was an increase of total power with gestational age which related temporally to changes in STV and SDNN, reaching significance at 9 days after baseline. Again, this increase was not observed in chronically hypoxic pregnancies, and there was an oxygen‐dependent difference between groups observed consistently from 9 days after baseline measurements (Fig. 4). There were no sustained changes of either gestational age or chronic hypoxia relating to total power in quiet sleep.

**Figure 5:**
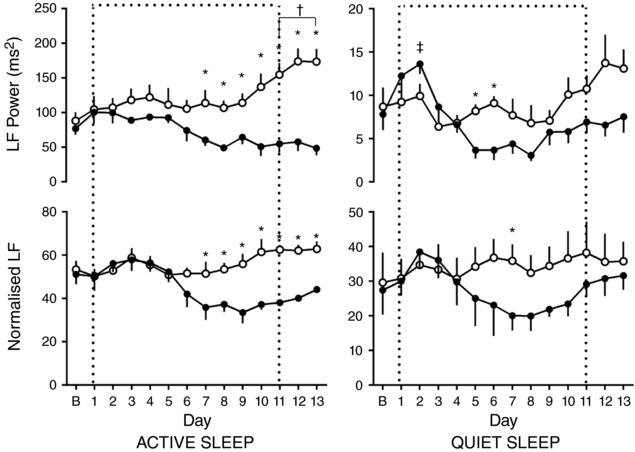
Ontogenic changes in indices of sympathetic contribution to fetal heart rate variability during chronic normoxia and hypoxia Values represent the mean ± SEM (*n* = 12) for absolute and normalised LF in quiet and active sleep. Period of chronic hypoxia (filled circles) or normoxia (open circles) indicated by dashed box. ‘B’ represents an average of values taken in the 72 h period −2 to 0. Significant differences (*P* < 0.05): ^*^significant effect of oxygenation between treatment groups; ^†^significant effect of gestational age in normoxic pregnancy on each individual day 1–13 when compared to the baseline ‘B’ period; ^‡^significant effect of gestational age in hypoxic pregnancy on each individual day 1–13 when compared to the baseline ‘B’ period (RM two‐way ANOVA with *post hoc* Tukey's and Holm–Sidak tests).

**Figure 6:**
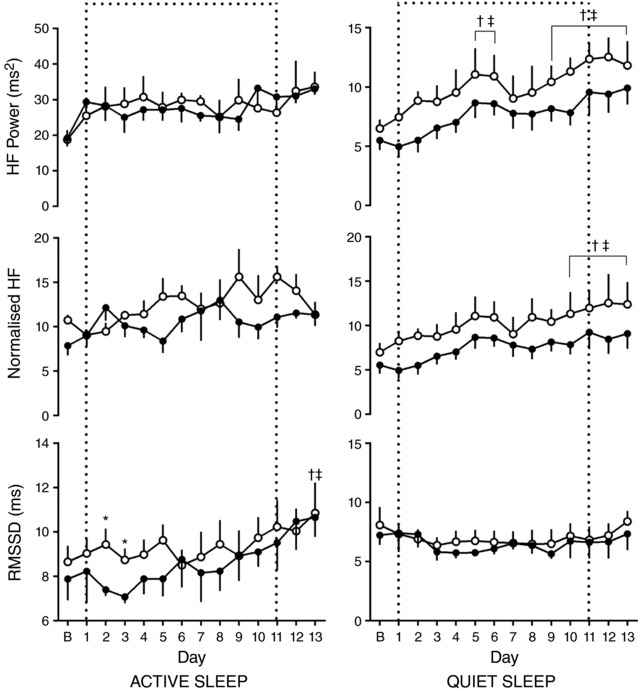
Ontogenic changes in indices of parasympathetic contribution to fetal heart rate variability during chronic normoxia and hypoxia Values represent the mean ± SEM (*n* = 12) for RMSSD, absolute and normalised HF in quiet and active sleep. Period of chronic hypoxia (filled circles) or normoxia (open circles) indicated by dashed box. ‘B’ represents an average of values taken in the 72 h period −2 to 0. Significant differences (*P* < 0.05): ^*^significant effect of oxygenation between treatment groups; ^†^significant effect of gestational age in normoxic pregnancy on each individual day 1–13 when compared to the baseline ‘B’ period; ^‡^significant effect of gestational age in hypoxic pregnancy on each individual day 1–13 when compared to the baseline ‘B’ period (RM two‐way ANOVA with *post hoc* Tukey's and Holm–Sidak tests).

In active sleep in normoxic pregnancy, there was a progressive increase in LF with increasing gestational age, which reached significance 11 days after baseline. However, when these values were related to increases in total power, there was no increase in normalised LF with gestation. In chronic hypoxia, after the initial response to the fall in oxygenation, there was a general trend for a decrease in both LF and normalised LF in active sleep. By 7 days after baseline, there was a significant difference in both LF and normalised LF in active sleep between normoxic and chronically hypoxic fetuses. This depression of LF and normalised LF in chronically hypoxic fetuses persisted to the end of the period of assessement (Fig. 5). There were no sustained differences seen in LF or normalised LF with gestational age or oxygenation seen in quiet sleep. Conversely, in quiet sleep there was a gestational age‐dependent increase in both HF and normalised HF observed from 9 and 10 days after baseline, respectively, in both normoxic and chronically hypoxic pregnancies, with no effect of treatment. This increase was not mirrored in RMSSD. There were no changes observed in parasympathetic indices of control of FHRV in active sleep (Fig. 6).

**Figure 7:**
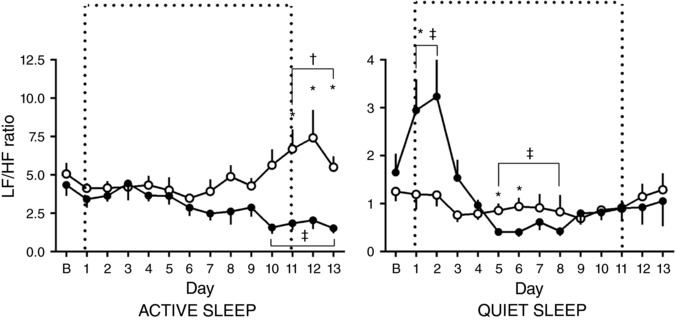
Ontogenic changes in sympathovagal balance during chronic normoxia and hypoxia Values represent the mean ± SEM (*n* = 12) for the LF to HF ratio in quiet and active sleep. Period of chronic hypoxia (filled circles) or normoxia (open circles) indicated by dashed box. ‘B’ represents an average of values taken in the 72 h period −2 to 0. Significant differences (*P* < 0.05): ^*^significant effect of oxygenation between treatment groups; ^†^significant effect of gestational age in normoxic pregnancy on each individual day 1–13 when compared to the baseline ‘B’ period; ^‡^significant effect of gestational age in hypoxic pregnancy on each individual day 1–13 when compared to the baseline ‘B’ period (RM two‐way ANOVA with *post hoc* Tukey's and Holm–Sidak tests).

In active sleep in normoxic pregnancy there was a gestational age‐related increase in the LF/HF ratio, which reached significance 11 days after baseline. Conversely, in hypoxic pregnancy there was a gestational age‐related fall in the LF/HF ratio, which reached significance at 10 days after baseline. Accordingly, there was an oxygenation‐dependent significant difference between groups evident 12 days after baseline. In quiet sleep, there was a significant shift to sympathetic dominance at the time of onset of hypoxia compared to normoxic pregnancies which shifts towards sympathetic suppression as the chronic hypoxia persists and an oxygenation effect measured from days 5 to 8 after baseline (Fig. 7).

## Discussion

This longitudinal study demonstrates that induction of late gestation chronic hypoxia has the potential to alter the development of autonomic nervous system control of FHRV in sheep, supporting the hypothesis tested. Exposure of pregnant ewes to a reduced fraction of inspired oxygen produced a sustained reduction in fetal femoral arterial $P_{O_{2}}$ to 10–12 mmHg between 0.8 and 0.9 of gestation. This represents an estimated 50% reduction in the normal oxygenation values of the fetal umbilical artery, 20 mmHg (Longo, 2013). Reductions of a similar or greater magnitude have been measured through sampling the umbilical vein in growth‐restricted human fetuses at comparable points in gestation (Soothill *et al*. 1987; Nicolaides *et al*. 1989). There was also a reduction in fetal arterial $P_{CO_{2}}$ in response to chronic hypoxia. This may suggest an alteration in the rate of fetal metabolism due to hypoxia, with a reduction in fetal oxygen consumption leading to a decrease in the production of fetal CO_2_ (see Allison *et al*. 2016). Therefore, the degree of chronic fetal hypoxia achieved in this study and the impact on fetal metabolism suggest this model is relevant to the human clinical situation in pregnancy complicated by significant fetal growth restriction.

In our study in normoxic fetuses there was an ontogenic increase in FHRV indices of overall variability (total power, SDNN) as well as an increase in STV with advancing gestation. The basal fetal heart rate also fell. These changes were more pronounced in active sleep but were still evident in quiet sleep. Total power has previously been shown to increase with advancing gestational age in fetal sheep (Koome *et al*. 2014a) and STV, measured by computerised CTG, has been observed to increase towards term in the human fetus (Serra *et al*. 2009). The mean STV values for our normoxic sheep fetuses fall between the 10th–50th centile of the published ranges for human fetuses at an equivalent gestational age, despite not being measured by computerised CTG in our fetuses (Serra *et al*. 2009). Therefore, our control group shows the expected ontogenic changes over the period studied, which represents approximately 10% of the total gestational period in sheep.

In the present study, exposure to chronic hypoxia prevented the ontogenic increase in FHRV indices of overall variability and STV, in both active and quiet sleep states, and accentuated the fall in basal fetal heart rate. Compared to their normoxic counterparts at an equivalent gestational age, STV, SDNN and total power were lower in chronically hypoxic fetuses, even after their return to normoxia. Despite this, the STV remained above 3 ms in hypoxic fetuses in active sleep states, which correlated well with the normal fetal arterial pH values observed throughout, and fetal survival to the end of the experimental procedure in all cases. Changes in cardiovascular function in these fetuses have been published previously (Allison *et al*. 2016). Chronic hypoxia diminished the ontogenic rise in fetal mean arterial blood pressure (fMAP) over the same period as this study, although the absolute difference by day 13 was <5 mmHg. There is a positive correlation between increases in mean arterial pressure and overall FHRV in the last third of gestation in fetal sheep, which is abolished by vagal blockade (Yu & Lumbers, 2000). However, in these studies, an increase in 10 mmHg of fMAP resulted in a <1% increase in overall FHRV (Yu & Lumbers, 2002). This is therefore unlikely to underlie the changes in FHRV or basal FHR seen.

Fetal breathing movements were not assessed in this study. In human fetuses, based on fMCG studies, fetal breathing episodes have been shown to increase both overall variability and HF power, and decrease basal heart rate (Gustafson *et al*. 2011). Furthermore, seminal studies have reported that chronic fetal hypoxia leads to a fall rather than an increase in the incidence of fetal breathing movements (Richardson *et al*. 1992). In fetal sheep, the effect of fetal breathing has been shown to account for less than 10% variation of the fetal heart rate, and changes predominantly result in a shift of power to HF, not an increase in total power, unlike the situation in the adult (Metsala *et al*. 1993). This is attributed to the small volume changes observed in the fetal lungs during *in utero* breathing compared to the adult tidal volume (McLain, 1964). Fetal breathing also produces a characteristic fetal heart rate pattern of respiratory sinus arrhythmia (pattern C) which correlates with fetal sleep state 3F, which should have been excluded from this study.

In human fetuses, impaired increases in overall heart rate variability with increasing gestational age, measured by computerised CTG, have been described in fetuses affected by uteroplacental dysfunction (Lobmaier *et al*. 2012). Similarly, fMCG studies have demonstrated a lower SDNN in human fetuses affected by uteroplacental dysfunction than was calculated in gestation age‐matched, normally grown controls (Sriram *et al*. 2013). Therefore, the data in our study highlight that changes in FHRV measured in human pregnancy affected by uteroplacental dysfunction are likely due to isolated chronic fetal hypoxia.

Both SDNN and STV are decreased in sympathectomised sheep compared to controls (Lear *et al*. 2016), as is total power (Koome *et al*. 2014a). Although resting sympathetic tone is low in the fetus (Assali *et al*. 1977), the sympathectomy studies suggest a significant role for the sympathetic nervous system in contributing to the ontogenic changes in FHRV. Sympathetic innervation is completed by the second half of pregnancy and increases gradually towards term in fetal sheep (Lebowitz *et al*. 1972). Conversely, the parasympathetic system, while active in the last third of gestation and exhibiting control over the FHR, does not begin to increase resting tone until term (van Laar *et al*. 2009). Consistent with the literature, in our normoxic fetuses, in active sleep, LF power, but not HF, normalised LF nor HF power, showed an increase with gestational age. The LF/HF ratio also increased with gestational age, suggesting an ontogenic shift towards sympathetic dominance as the healthy fetus approached term. Compared to normoxic fetuses, the LF and normalised LF power was significantly lower in chronically hypoxic fetuses in active sleep, while HF and normalised HF power remained unchanged. This resulted in an ontogenic decrease rather than increase in LF/HF ratio, and a marked difference in LF/HF ratio between normoxic and chronically hypoxic fetuses by the end of the experimental protocol. This appeared to be due to a reduction in the influence of the sympathetic nervous system on the control of FHRV, rather than due to changes in the parasympathetic nervous system in the chronically hypoxic fetus. As previously described, the impaired ontogenic increase in mean arterial pressure in the chronically hypoxic fetuses (Allison *et al*. 2016) would not be expected to affect the increase of power in the LF spectrum, or promote a shift toward sympathetic domination (Yu & Lumbers, 2002). In theory, due to the impaired rise in mean arterial blood pressure, an increase in HF power could have been expected, but this was not seen. This is likely due to the small, but significant, absolute difference in fetal mean arterial blood pressure between the normoxic and chronically hypoxic fetuses. This reduction in the influence of the sympathetic nervous system could also underlie the change in basal heart rate in the chronically hypoxic fetus.

The chronically hypoxic fetuses were, at the start of the experimental protocol, capable of increasing the sympathetic control of FHRV in response to an acute challenge. There was an increase in LF, normalised LF and the LF/HF, seen in quiet sleep, during the first 48 h of exposure to hypoxia, representing a prolonged period of sympathetic dominance in the control of FHRV. This increase in sympathetic nervous output is a characteristic response to acute hypoxia, and forms part of the fetal cardiovascular defence mechanism to hypoxic episodes (Giussani, 2016). However, the increased sympathetic tone cannot be maintained indefinitely, and blunting of fetal heart rate and peripheral vascular responses to sympathetic activity has been reported after around 72 h (Bennet & Gunn, 2009). In our animals, it is only after this time that the reduction in sympathetic contribution of FHRV becomes evident. This likely underlies the delay in onset of the fall in basal FHR seen in chronically hypoxic fetuses. Therefore, unlike the normoxic fetuses, in which the trend was to develop sympathetic dominant control of FHRV approaching term, the trend in the chronically hypoxic fetus was towards overall sympathetic suppression, despite a normal ontogenic rise in fetal plasma cortisol levels. The fetal endocrine data are in keeping with studies of ovine pregnancy at high altitude. Such studies have reported that ovine fetuses exposed to chronic hypoxia have unaltered levels of basal cortisol and catecholamines compared to normoxic controls at corresponding gestational ages (Newby *et al*. 2015). This study, however, was not able to suggest the mechanism by which this sympathetic suppression occurred in chronically hypoxic fetuses, given that no changes in fetal basal cortisol or catecholamine levels were detected, and no other assessment of sympathetic function or innervation was performed.

The findings reported in the present study agree with fMCG studies, which show a progressive increase in LF power in normoxic human pregnancy (Ferrario *et al*. 2009; Schneider *et al*. 2010). The LF/HF ratio in human pregnancies also shows an ontogenic increase and a change towards sympathetic dominance during the third trimester (Schneider, 2009; Fukushima *et al*. 2011), while HF power remains unchanged (Ferrario *et al*. 2009). Lower values of LF/HF ratio are associated with decreased overall FHRV (Ferrario *et al*. 2009) and STV (Schneider *et al*. 2010). In human fetuses in pregnancy complicated by uteroplacental dysfunction, overall FHRV is reduced due to a reduction in the sympathetic contribution to the control of the fetal heart, while the parasympathetic contribution remains unchanged (Ferrario *et al*. 2009; Sriram *et al*. 2013). A decrease in LF has been linked to an umbilical artery $P_{O_{2}}$ < 20 mmHg at delivery (Ohta *et al*. 1999; Suzuki *et al*. 2000). Our studies again highlight that sympathetic suppression in the control of FHRV in the late gestation human fetus in complicated pregnancy is likely due to isolated chronic hypoxia. Collectively, past and present data suggest that a reduction in overall FHRV in the human fetus, as in fetal sheep, may be due to sympathetic suppression rather than an increase in parasympathetic tone. Appropriate activation of the sympathetic nervous system in the late gestation fetus is an indispensable component of the fetal homeostatic compensatory response to acute stress, such as in response to acute reductions in fetal oxygenation or acute hypoxia (Giussani *et al*. 1993; Giussani, 2016). Therefore, dysregulation of sympathetic control of the cardiovascular system would predict that the chronically hypoxic fetus may be more vulnerable to acute insults in late gestation. Reduction in overall FHRV, or STV, may therefore provide a biomarker that autonomic dysregulation of FHR control has taken place in a fetus where uteroplacental dysfunction is suspected. This study does not, however, suggest the mechanism by which this dysregulation of sympathetic control occurs in the chronically hypoxic fetus.

In conclusion, we present the first longitudinal study of autonomic control of fetal heart rate variability in isolated chronic fetal hypoxia. We have demonstrated that FHRV in sheep is reduced in chronic hypoxia, predominantly due to dysregulation of the sympathetic control of the fetal heart rate. This presents a potential mechanism by which a reduction in indices of FHRV in human fetuses predicts an increased risk of birth asphyxia.

## Additional information

### Competing interests

D.A.G. worked with Maastricht Instruments to design and create the data acquisition system and with Telstar ACE to design the hypoxic chambers. No other authors have competing interests.

### Disclosure

License agreement 100395 CamDAS: technology for simultaneous wireless recording of arterial blood pressure and blood flow in large animals. D. A. Giussani, Maastricht Instruments, the British Heart Foundation and Cambridge Enterprise.

### Author contributions

The experiments in this study were performed in the Department of Physiology, Development and Neuroscience, University of Cambridge. D.A.G., C.C.L., C.J.S., B.J.A. and Y.N. conceived and designed the experiments. D.A.G., C.J.S., B.J.A., Y.N., K.J.B. and N.I. collected, analysed and interpreted the experimental data. C.J.S., D.A.G. and C.C.L. drafted the article and all authors contributed to revising it for important intellectual content. All authors have approved the final version of the manuscript and agree to be accountable for all aspects of the work. All persons designated as authors qualify for authorship, and all those who qualify for authorship are listed.

### Funding

This work was supported by the British Heart Foundation and Action Medical Research (GN2052).
